# Rapidly Growing Chondroid Syringoma of the External Auditory Canal: Report of a Rare Case

**DOI:** 10.1155/2011/589680

**Published:** 2011-09-15

**Authors:** Ioannis Vasileiadis, Stylianos Kapetanakis, Aristotelis Petousis, Euthimios Karakostas, Christos Simantirakis

**Affiliations:** ^1^Department of Otolaryngology/Head and Neck Surgery, Venizeleio-Pananeio Hospital, Knossou Boulevard, 71409 Herakleion, Greece; ^2^Department of Anatomy, Medical School of Alexandroupolis, Democritus University of Thrace, University Hospital of Alexandroupolis, 68100 Alexandroupolis, Greece

## Abstract

*Introduction*. Chondroid syrinoma of the external auditory canal is an extremely rare benign neoplasm representing the cutaneous counterpart of pleomorphic adenoma of salivary glands. Less than 35 cases have been reported in the international literature. *Case Presentation*. We report a case of a 34-year-old male in whom a rapidly growing, well-circumscribed tumor arising from the external auditory canal was presented. Otoscopy revealed a smooth, nontender lesion covered by normal skin that almost obstructs the external auditory meatus. MRI was performed to define the extension of the lesion. It confirmed the presence of a 1.5 × 0.8 cm T2 high-signal intensity lesion in the superior and posterior wall of EAC without signs of bone erosion. The patient underwent complete resection of the tumor. The diagnosis was confirmed by histopathologic examination. *Conclusion*. Although chondroid syringoma is extremely rare, it should always be considered in the differential diagnosis of an aural polyp. Chondroid syringomas are usually asymptomatic, slow-growing, single benign tumors in subcutaneous or intradermal location. In our case, the new information is that this benign tumor could present also as a rapidly growing lesion, arising the suspicion for malignancy.

## 1. Introduction

Chondroid syringoma or mixed tumor of skin is a benign neoplasm of sweat gland origin. It arises most frequently in the nose, cheek, scalp, forehead, upper lip, and chin. Chondroid syringomas of the external auditory canal are extremely rare. Less than 35 cases have been reported in the international literature. All authors agree that the tumors in this location derive from the ceruminous glands in the skin of the external auditory canal. The tumor is usually presented in patients between 20 and 60 years as a slowly growing, encapsulated, painless subcutaneous mass. In the case reported here, the tumor presented as rapidly growing solid mass in the external auditory canal of a young man. We discuss the diagnosis, differential diagnosis of this mass, and the relevant literature.

## 2. Case Report

**Figure 1:**
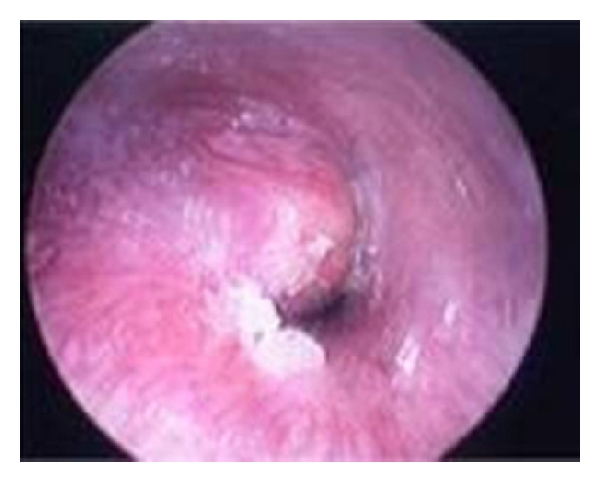
Endoscopic view of the smooth mass of the posterior wall of the right external auditory canal.

**Figure 2:**
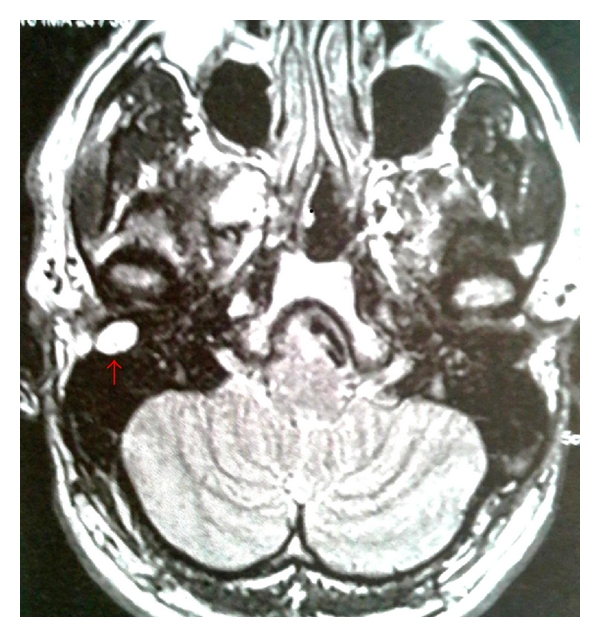
Axial T2-weighted MRI image. A well-bordered lesion was detected in the right external auditory canal. After intravenous gadolinium injection, the lesion had high signal intensity on T2-weighted image.

**Figure 3:**
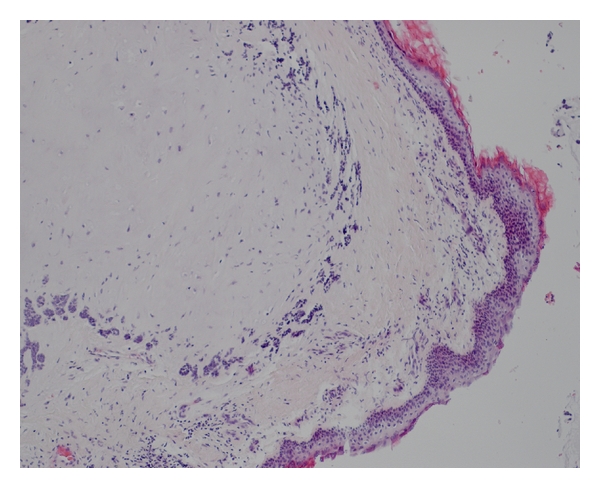
Histological appearance of the tumor showing the epithelial part under dense collagen tissue (H & E  ×40).

**Figure 4:**
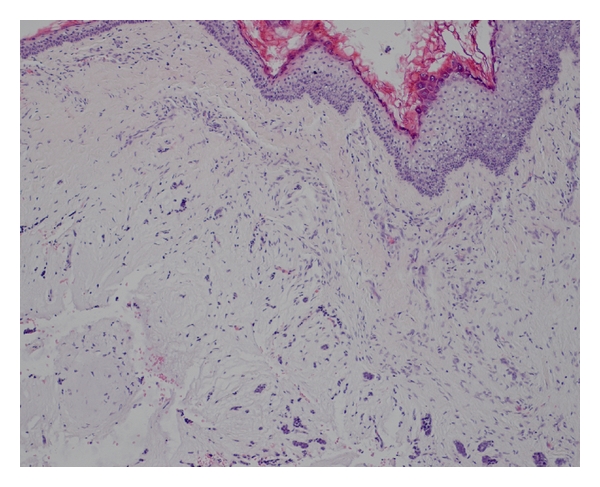
Histological appearance of the tumor showing ducts and tubules focally observed (H & E  ×100).

**Figure 5:**
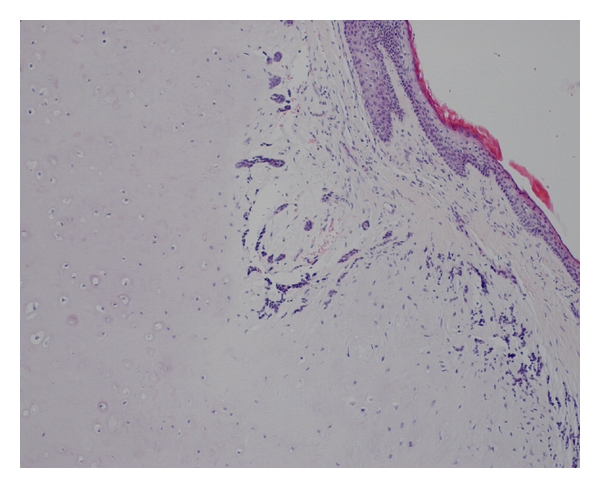
Histological appearance of the tumor showing the stroma of myxoid matrix (H & E  ×100).

A 34-year-old Caucasian man, resident doctor, was referred to our Department of Otolaryngology with the complaint of a painless mass in the posterior wall of the right EAC associating with a reduction in hearing. He has been noticed the lesion three months ago. The otoscopy revealed a smooth, nontender lesion covered by normal skin that almost obstructs the external auditory meatus (Figure 1). The threshold tonal audiometry confirmed a transmission hearing loss in right ear. An MRI was performed to define the extension of the lesion. It confirmed the presence of a 1.5 × 0.8 cm T2 high-signal intensity lesion in the superior and posterior wall of EAC without signs of bone erosion (Figure 2). The lesion was excised surgically, and the histopathologic examination revealed a chondroid syringoma (Figures 3, 4, and 5). The patient is followed up regularly, and there is no evidence of recurrence 15 months after the operation.

## 3. Discussion

Chondroid syringoma is a rare mixed tumor of the skin that was first described by Hirsch and Helwig in 1961 [1]. It is a benign neoplasm of sweat gland origin that occurs most frequently in the head and neck region. The commonest sites are the scalp, cheek, nose, upper lip, chin, and the forehead. Extremely rare sites include eyelid, orbit, and scrotum [2]. Chondroid syringoma arising from the EAC is also extremely rare. Only 35 cases have been reported in the English literature [1–3].

Typically, tumor presents in male patients between 20 and 60 years as a slowly growing, painless, subcutaneous mass [2]. In our case, the tumor was presented only three months before the diagnosis, and it was rapidly growing. The patient was a resident doctor, and we could consider his medical history reliable.

The diagnosis is usually made retrospectively based on histopathological findings. Mixed tumor of skin histopathologically consists of tubuloductal epithelial structures of sweat gland origin and is associated with chondroid changes [4]. Differential diagnosis should include basal cell carcinoma, squamous cell carcinoma, dermatofibroma, neurofibroma, histiocytoma, implantation dermoid, and sebaceous cyst [3, 4]. Although chondroid syringomas are predominantly benign, malignant forms have been reported [5].

Although chondroid syringomas of external auditory canal are slowly growing tumors, in this case, the rapid growing of the skin lesion raised the suspicion of a malignancy. Therefore, surgical excision and histopathological examination were compulsory to exclude this possibility. 

Preoperative evaluation by MRI is superior to that by CT, enabling differential diagnosis of other tumors of EAC and indicates possible erosion in the bony tissue [6]. For cases that no erosion of the bone is indicted, surgical excision is the treatment of choice. A complete resection is required to prevent recurrence [5, 6].

## 4. Conclusion

Chondroid syringoma should be included in the differential diagnosis of small subcutaneous nodules in the EAC, in middle-aged male patients. Although malignant forms and recurrence are extremely rare, wide excision of the tumor and long-term followup are the most effective ways of management.

## Abbreviation

EAC: External auditory canal.
